# Impact of COVID-19 on the management of hypertension: a perspective on disease severity, service use patterns and expenditures from Ghana’s health insurance claims data

**DOI:** 10.1038/s41371-024-00924-3

**Published:** 2024-06-20

**Authors:** Ama Pokuaa Fenny, Evans Otieku, Samuel Owusu Achiaw, Bernard Okoe Boye, Francis Asenso-Boadi, Vivian Addo-Cobbiah, Mariam Musah

**Affiliations:** 1 Institute of Statistical, Social, and Economic Research (ISSER), University of Ghana, Accra, Ghana; 2 Department of Public Health, Aarhus University, Aarhus, Denmark; 3 Health Economics and Health Technology Assessment, School of Health and Wellbeing, University of Glasgow, Scotland, United Kingdom; 4 National Health Insurance Authority, Accra, Ghana

**Keywords:** Hypertension, Health care

## Abstract

Hypertension is a leading cause of morbidity in Ghana and other sub-Saharan African countries, but management has historically suffered from the fragility of health systems in these countries. This has been exacerbated by the COVID-19 pandemic and its associated measures. Our study examines and quantifies the effect of the pandemic on the management of hypertension in Ghana by determining changes in disease severity and presentation, as well as changes in health service use patterns and expenditures. We used cross-sectional data to perform an impact evaluation of COVID-19 on hypertension management before and during the pandemic. We employed statistical tests including t-tests, z-tests, and exact Poisson tests to estimate and compare hypertension episode intensity and related claim expenditures before and during the pandemic using medical claims data from Ghana’s National Health Insurance Authority database. The study duration includes a 12-month reference/pre-pandemic period (March 2019–February 2020) relative to the target/pandemic period (March 2020–February 2021). We observed that although there was a 20% reduction in the number of hypertension claimants in the pandemic year, there was an increase in hypertension severity as measured by the number of hypertension episodes per claimant. There was also an 18.64% or $22.88 (95% CI: $21–$25, *p* = 0.01042) increase in the average cost per hypertension claimant in the pandemic year. The increase in episodes per claimant had the largest financial impact on the average cost per claimant. The findings from our studies are relevant for future policymaking and strategy implementation for hypertension control in Ghana.

## Introduction

Ghana, like most of sub-Saharan Africa (SSA), faces a double burden of disease [1]. While infectious diseases like malaria have always been of prime importance, with the epidemiological transition in SSA, non-communicable diseases (NCDs) have increasingly become leading causes of mortality and morbidity [2]. It has been projected that by 2030, NCDs will be the foremost cause of death in the region exceeding deaths from communicable, maternal, perinatal, and nutritional diseases [3]. In 2015, NCDs accounted for a productivity loss of over Int$1.1 trillion in Africa, the highest for any disease group measured [4, 5]. Cardiovascular diseases (CVDs) such as coronary heart disease and stroke account for the majority of NCD-related deaths [6]. The leading modifiable risk factor for these CVDs is (primary) hypertension which has its highest prevalence in African countries [7, 8]. In Ghana, for instance, a meta-analysis by Atibila et al. [9] reported the prevalence of hypertension in the general population to be 30%, rising to approximately 44% in the adult population. Additionally, hypertension has been recognised as the leading cause of disability among Ghanaian adults [10].

The double burden of disease has put a strain on the limited healthcare resources in Ghana and, given the historical epidemiological dominance of infectious diseases, the allocation of healthcare resources in the country has been and continues to be disproportionate with less focus being placed on hypertension and other NCDs despite their increasing relevance [2]. That is to say, healthcare resource allocation in the country has failed to correspondingly evolve with the epidemiology. Data from the Ghana national health accounts published in 2017 showed that of the over GH₵8 billion spent on healthcare in 2015, only 12.6% was spent on NCDs compared to approximately 40.8% on infectious diseases [11]. The same trend was reported over the previous 2 years [11]. Consequently, access to healthcare for NCDs has been inadequate; Kushitor et al. [12] have reported that only 35% of healthcare facilities in Ghana have essential drugs for diabetes and hypertension.

It was against this backdrop that Ghana was hit by the COVID-19 pandemic. The WHO reported that the pandemic and its associated measures resulted in the disruption and, in some cases, the collapse of essential health services in almost all countries (about 90%); however, these disruptions were more severe in low- and middle-income countries (LMICs) [13]. African countries were some of the most affected with the disruption of a median of 60% (up to 90% in some African countries) of all essential health services including those for infectious diseases; maternal, reproductive, and child health; emergency care; as well as those for non-communicable diseases [13]. The disruption of health services is particularly important in the case of NCDs as these diseases not only require long-term care but can progress into advanced stages and result in severe complications without showing significant symptoms [14, 15]. In some cases, such complications may be the first manifestation of the disease [14, 15]. For hypertension, also referred to as the “silent killer”, some patients may first present with major cardiovascular events such as strokes or myocardial infarctions. Even for those diagnosed, poor management may result in these cardiovascular complications and others such as kidney failure [16, 17]. Disruption of healthcare for hypertension leads to delayed or forgone care which results in a significant increase in mortality and morbidity and, resultingly, an extortionate social and economic toll. It has been globally acknowledged that these pandemic-related health service disruptions would have long-term effects on health systems and population health, necessitating research to understand the impact of these disruptions, particularly in LMICs like Ghana where systems were already fragile [13].

There is, however, a dearth of research in Ghana investigating the impact of COVID-19 and its associated measures on the management of hypertension. Given the economic and disease burden of hypertension, especially those likely to result from complications due to disruptions in care, it is necessary to quantify the impact of COVID-19 on the management of hypertension to inform policymakers and other relevant stakeholders. Using claims data from the Ghana National Health Insurance Authority (NHIA), this study investigates the impact of the pandemic on the management of hypertension by identifying the changes in disease severity, health service use patterns, and the resulting changes in expenditures for the condition.

## Materials and methods

### Design, sampling, and grouping

We used cross-sectional data to perform an impact evaluation of COVID-19 on disease severity, health service use patterns, and expenditures for hypertension treatment before and during the pandemic. Participants comprised all age groups registered under the National Health Insurance Scheme (NHIS), and whose insurance claims were submitted to the NHIA by accredited healthcare providers for treatment of hypertension 12 months before (3/March/2019–2/February/2020) and during (3/March/2020–2/February/2021) the outbreak of COVID-19. The NHIA is a health regulatory authority established by law to, among other things, provide financial risk protection to enhance universal access to quality health care services by all residents in Ghana [18]. The authority receives, processes, and pays for health insurance claims routinely submitted by accredited health care service providers across the sixteen geographic regions of Ghana [18]. For this study, we focused on claim expenditures and service use patterns in three geographic regions of Ghana, that is, Greater Accra, Ashanti, and Northern regions to represent the coastal, middle, and northern belts of Ghana, respectively.

We studied about 2 million hypertension episode claims from the NHIA database. The data includes claims for medical and pharmaceutical expenses for individuals on the NHIS diagnosed with hypertension by qualified healthcare professionals following clinical guidelines [19, 20]. For adults, hypertension was defined as persistently having systolic blood pressure (SBP) values ≥ 140 mmHg and/or diastolic blood pressure (DBP) values ≥ 90 mmHg [19, 20]. For children (<18 years), hypertension was defined as an average SBP and DBP values ≥ 95th percentile for gender, weight, and height from 3 appropriately taken blood pressure readings [19]. The NHIA classified all claims related to hypertension into episode groups (group 1 – chronic follow-up due to hypertension, and group 2 – emergency/acute hypertension) using the Ghana Diagnostic Related Groupings (G-DRG). Chronic follow-up episodes referred to outpatient appointments for hypertension and emergency/acute episodes referred to emergency attendance or inpatient treatment of hypertension.

This study only considered claims related to primary chronic hypertension, i.e. hypertension for which no underlying cause has been identified [19]. Primary hypertension was indicated in the diagnosis description in the claims data; however, since primary hypertension is commonly referred to as just “hypertension”, any claim with a diagnosis of “hypertension” without qualifiers such as “secondary”, “pregnancy-induced”, “gestational” “hypertension in pregnancy”, or “ocular” was assumed to be a claim for primary hypertension.

In this study, a claim corresponds to an episode and these terms were used interchangeably.

### Outcome measurement

Outcome measures were changes in hypertension severity, healthcare service use patterns, and the extra claim costs (financial impact) observed during COVID-19.

Firstly, the total cost of all hypertension episodes, the average/mean cost per episode (that is the average cost when both follow-up and emergency/acute episodes are considered together), the average cost per follow-up episode, the average cost per emergency acute episode, and the average cost per claimant were estimated for the pre-pandemic and then the pandemic year.

Following this, changes in health service use patterns for hypertension due to the pandemic were measured as:

1. Follow-up episode ratios (FERs) and emergency/acute episode ratios (EER) before and during the pandemic.
2. Emergency/acute episode to follow-up episode ratio (EFER).
3. Episode per claimant rate (ECR) or the episode intensity.

Where:

- Follow-up episode ratio (FER) = Number of follow-up episodes/Number of all primary hypertension episodes or claims.
- Emergency/acute episode ratio (EER) = Number of all emergency episodes/Number of all primary hypertension episodes or claims.
- Episode per claimant rate (ECR) = Total number of claims or episodes/ Total number of claimants.
- Emergency/acute episode to follow-up episode ratio (EFER) = Number of emergency acute episodes/Number of follow-up episodes.

ECR and EER were proxy measures for hypertension severity, with the assumption that an increase in severity would lead to an increase in emergency/acute presentations (hence an increase in EER) and require multiple episodes of care per patient (hence an increase in ECR).

The financial impact of the change in service use patterns (ECR, EER, and FER) on the cost of management for hypertension (average cost per claimant) was measured.

The ratios are related to the average cost per claimant by the formulae below:

1. Average cost per claimant (ACPC) = average cost per episode (ACPE) × ECR.
2. Average cost per episode (ACPE) = (FER × Average cost per follow-up episode) + (EER x Average cost per emergency/acute episode).
3. Average cost per claimant (ACPC) = ECR × ((FER × Average cost per follow-up episode) + (EER × Average cost per emergency/acute episode)).

The financial impact of the various factors (i.e., ECR, FER, and EER) was defined as the difference in the average cost per claimant (ACPC) when that factor or ratio takes on the value in the pandemic period while other variables in the equation remain the same for the pre-pandemic or reference year. For example, the financial impact of the change in ECR was calculated as:

$$\left({{{{{{\rm{ACPE}}}}}}}_{{{{{{\rm{pre}}}}}}-{{{{{\rm{pandemic}}}}}}}\,{{{{{\rm{x}}}}}}\,{{{{{{\rm{ECR}}}}}}}_{{{{{{\rm{pandemic}}}}}}}\right)-{{{{{{\rm{ACPC}}}}}}}_{{{{{{\rm{pre}}}}}}-{{{{{\rm{pandemic}}}}}}}$$

The calculation of the financial impact follows the methodology used by Li et al. [21]. who studied the impact of COVID-19 on treatment for mental health conditions. Other aspects of this study’s methods including the calculation of the ECR were adapted from Li et al.’s [21] study.

### Statistical analysis

Independent t-tests were used to estimate the differences in mean cost per hypertension episode and claimant pre-pandemic and during the pandemic. Independent t-tests were deemed appropriate as the analysis excluded 819 individuals with paired data (observations in both the pre-pandemic and pandemic years) using unique NHIS identity codes. The differences in ratios (FER and EER) were estimated with two proportions z-tests. Exact Poisson tests were used to measure the ratio/difference in ECR in the pre-pandemic and pandemic years. The exact Poisson test measures the difference or ratio between Poisson rates for two independent samples [22]. Test estimates were accompanied by 95% confidence intervals (95%CI) and the analysis assumed a normal distribution in hypertension claims expenditures for each episode group. The result of a test was of statistical significance if *p* < 0.05.

We performed all expenditure analyses considering the age group of claimants and reported the endpoint financial impact of COVID-19 on hypertension care in Ghana in 2021 purchasing power parity in international United States Dollars using a recommended standard web-based calculator [23]. The analysis was done in Microsoft® Excel® for Microsoft 365 MSO (Version 2404 Build 16.0.17531.20004), Stata Version 16, and R version 4.3.2.

## Results

### Description of service use patterns

For the pre-pandemic year, there were 1,074,611 hypertension episodes by 291,385 claimants from 578 accredited facilities, and for the pandemic year, there were 1,011,212 episodes by 233,636 claimants from 544 accredited facilities. Most accredited facilities that submitted claims for hypertension were primary care facilities with the Ashanti region having the highest number of accredited facilities submitting claims for both years. See Table 1 for the distribution of accredited facilities by type and geographic region.

**Table 1:** Distribution of accredited healthcare facilities that submitted claims to the NHIS by facility type and geographic region.

	Before pandemic	During pandemic
Facility type	Number of facilities	Number of facilities
CHPS Compound	44	38
Clinic	87	76
Health Centre	18	12
Polyclinic	24	21
Primary Care Hospital	325	324
Secondary Care Hospital	44	37
Tertiary Care Hospital	36	36
Total	578	544
Geographic region		
Ashanti	274	251
Greater Accra	142	136
Northern	162	157
Total	578	544

For the pandemic year, there were 72,512 chronic follow-up episodes/claims, a 13.6% drop from 83,962 episodes recorded in the pre-pandemic year. There was also a 5.2% drop in the number of emergency/acute episodes in the pandemic year. Generally, for both years, the number of hypertensive episodes increased with age with the peak number of episodes recorded for claimants aged 55–64 years. Further details are outlined in Table 2.

**Table 2:** Number of claimants, episodes for hypertension, and episode-to-claimant rate (ECR) by age groups.

	Pre-pandemic year (03/19–02/20)			Pandemic year (03/20–02/21)				Changes observed in pandemic year		
*Age group*	*Number of claimants per year*	*Estimated annual episodes*	*Episode- to- claimant rate (ECR) (95% CI)*	*Number of claimants per year*	*Estimated annual episodes*	*ECR (95% CI)*	Rate ratio (95% CI, p-value*)	*% change in number of claimants*	*% change in number of annual episodes*	*% change in estimated mean annual ECR*
*CFU*										
0–4 years	40	126	3.15 (2.62–3.75)	16	69	4.31 (3.36–5.46)	1.37 (1.01–1.85, *p* = 0.039)	−60.00%	−45.24%	36.90%
5–14 years	8	27	3.38 (2.22–4.91)	11	44	4 .00 (2.91–5.37)	1.19 (0.72–1.99, *p* = 0.549)	37.50%	62.96%	18.52%
15–24 years	79	260	3.29 (2.90–3.72)	86	345	4.01 (3.60–4.46)	1.22 (1.03–1.44, *p* = 0.016)	8.86%	32.69%	21.89%
25–34 years	433	1569	3.62 (3.45–3.81)	399	1722	4.32 (4.11–4.52)	1.19 (1.11–1.28, *p* < 0.001)	−7.85%	9.75%	19.10%
35–44 years	1927	6454	3.35 (3.27–3.43)	1494	6169	4.13 (4.03–4.23)	1.23 (1.19–1.28, *p* < 0.001)	−22.47%	−4.42%	23.29%
45–54 years	4205	16,316	3.88 (3.82–3.94)	3392	14,248	4.20 (4.13–4.27)	1.08 (1.06–1.11, *p* < 0.001)	−19.33%	−12.67%	8.26%
55–64 years	5943	22,879	3.85 (3.80–3.90)	4329	19,957	4.61 (4.55–4.67)	1.20 (1.17–1.22, *p* < 0.001)	−27.16%	−12.77%	19.75%
65–74 years	4874	18,570	3.81 (3.76–3.87)	3444	15,186	4.41 (4.34–4.48)	1.16 (1.13–1.18, *p* < 0.001)	−29.34%	−18.22%	15.73%
>74 years	4496	17,761	3.95 (3.90–4.00)	3015	14,772	4.90 (4.82–4.98)	1.24 (1.21–1.27, *p* < 0.001)	−32.94%	−16.83%	24.03%
*Sub-total*	*22,005*	*83,962*	*3.82 (3.79–3.84)*	*16,186*	*72,512*	*4.48 (4.45–4.51)*	*1.17 (1.16–1.19, p* < *0.001)*	*−26.44%*	*−13.64%*	*17.41%*
*EAE*										
0–4 years	308	1131	3.67 (3.46–3.89)	280	1166	4.16 (3.93–4.41)	1.13 (1.04–1.23, *p* = 0.002)	−9.09%	3.09%	13.40%
5–14 years	281	1041	3.7 (3.48–3.94)	141	610	4.33 (3.99–4.68)	1.17 (1.06–1.29, *p* = 0.002)	−49.82%	−41.40%	16.78%
15–24 years	1610	5489	3.41 (3.32–3.50)	1134	4536	4 (3.88–4.12)	1.17 (1.13–1.22, *p* < 0.001)	−29.57%	−17.36%	17.33%
25–34 years	7901	28,050	3.55 (3.51–3.59)	6587	27,796	4.22 (4.17–4.27)	1.19 (1.17–1.21, *p* < 0.001)	−16.63%	−0.91%	18.86%
35–44 years	25,720	91,049	3.54 (3.52–3.56)	17,871	85,066	4.76 (4.73–4.79)	1.34 (1.33–1.36, *p* < 0.001)	−30.52%	−6.57%	34.46%
45–54 years	52,640	197,399	3.75 (3.73–3.76)	43,142	185,943	4.31 (4.29–4.33)	1.15 (1.14–1.16, *p* < 0.001)	−18.04%	−5.80%	14.93%
55–64 years	75,948	270,375	3.56 (3.55–3.57)	61,547	257,884	4.19 (4.17–4.21)	1.18 (1.17–1.18, *p* < 0.001)	−18.96%	−4.62%	17.70%
65–74 years	54,906	199,857	3.64 (3.62–3.66)	45,868	192,644	4.20 (4.18–4.22)	1.15 (1.15–1.16, *p* < 0.001)	−16.46%	−3.61%	15.38%
>74 years	50,066	196,258	3.92 (3.90–3.94)	40,320	183,055	4.54 (4.52–4.56)	1.16 (1.15–1.17, *p* < 0.001)	−19.47%	−6.73%	15.82%
*Sub-total*	*269,380*	*990,649*	*3.68 (3.67–3.68)*	*216,890*	*938,700*	*4.33 (4.32–4.33)*	*1.18 (1.17–1.18, p* < *0.001)*	*−19.49%*	*−5.24%*	*17.69%*
Grand total	291,385	1,074,611	3.69 (3.68–3.69)	233,076	1,011,212	4.34 (4.33–4.34)	1.18 (1.17–1.18, *p* < 0.001)	−20.01%	−5.90%	17.64%

*CFU* Chronic follow–up episode, *EAE* Emergency Acute Episode.

*p-values for Exact Poisson Test comparing pre-pandemic ECR with pandemic ECR.

The overall cost for all primary hypertension episodes in the pandemic year was $33,957,143.20 compared to $35,783,849.99 in the pre-pandemic year. A significant proportion of these total costs were due to emergency/acute episodes for both years (95.3% for the pre-pandemic year and 95.7% for the pandemic year). Table 3 shows the ACPE by type of hypertension episode and stratified by age group. For the pre-pandemic year, the ACPE was $33.30 compared to $33.58 in the pandemic year. The ACPC in the pandemic year was $145.69, representing 18.64% or $22.88 (95% CI: $21–$25, *p* = 0.01042) increase from the pre-pandemic year.

**Table 3:** Costs for the management of hypertension in the pre-pandemic and pandemic years.

	Pre-pandemic year			Pandemic year			Changes observed in the pandemic year		
*Age group*	*Number of episodes per year*	*ACPE by age group (USD) [95% CI]*	*Total cost of episodes by age group [USD]*	*Number of episodes per year*	*ACPE by age group (USD)*	*Total cost of episode by age groups [USD]*	*% change in mean cost*	*% change in total cost by age groups*	*p*-value*
*CFU*									
0–4 years	126	17.5 [15.3–19.7]	2205.00	69	17.55 [9.40–25.56]	1210.95	0.29%	−45.08%	0.1057
5–14 years	27	20.18 [13.1–27.26]	544.86	44	20.21 [13.51–26.91]	889.24	0.15%	63.21%	0.1041
15–24 years	260	24.49 [19.7–29.28]	6367.40	345	24.56 [21.03–28.09]	8,473.20	0.29%	33.07%	0.125
25–34 years	1569	18.72 [17.11–20.33]	29,371.68	1722	18.75 [17.16–20.34]	32,287.50	0.16%	9.93%	0.203
35–44 years	6454	18.71 [17.56–19.86]	120,754.34	6169	18.79 [17.30–20.28]	115,915.51	0.43%	−4.01%	0.249
45–54 years	16,316	19.36 [18.77–19.95]	315,877.76	14248	19.41 [18.05–20.77]	276,553.68	0.26%	−12.45%	0.201
55–64 years	22,879	20.18 [19.52–20.84]	461,698.22	19957	20.25 [19.11–21.39]	404,129.25	0.35%	−12.47%	0.139
65–74 years	18,570	20.57 [19.08–22.06]	381,984.90	15186	20.7 [19.41–21.99]	314,350.20	0.63%	−17.71%	0.102
>74 years	17,761	19.93 [18.71–20.61]	353,976.73	14772	20.07 [19.72–20.42]	296,474.04	0.70%	−16.24%	0.171
Sub-total	83,962		1,672,780.89	72,512		1,450,283.57		−13.30%	
Average/mean cost of CFU episode		19.92			20		0.39%		0.1431
		[17.67–22.17]			[17.11–22.89]				
Average cost per claimant for CFU		76.02			89.6		17.87%		0.01063
		[73.20–78.84]			[84.51–94.69]				
EAE									
0–4 years	1131	29.66 [23.07–36.25]	33,545.46	1166	29.77 [26.05–33.44]	34,711.82	0.37%	3.48%	0.1092
5–14 years	1041	27.67 [23.41–31.93]	28,804.47	610	27.82 [25.17–30.47]	16,970.20	0.54%	−41.08%	0.1038
15–24 years	5489	43.93 [39.07–48.79]	241,131.77	4536	43.96 [39.10–48.82]	199,402.56	0.07%	−17.31%	0.106
25–34 years	28,050	41.47 [38.41–44.53]	1,163,233.50	27796	41.48 [38.13–44.83]	1,152,978.08	0.02%	−0.88%	0.3041
35–44 years	91,049	37.25 [35.81–38.69]	3,391,575.25	85066	37.32 [34.71–39.93]	3,174,663.12	0.19%	−6.40%	0.1072
45–54 years	197,399	34.56 [33.07–36.05]	6,822,109.44	185943	34.67 [33.07–36.05]	6,446,643.81	0.32%	−5.50%	0.1551
55–64 years	270,375	33.6 [32.03–35.17]	9,084,600.00	257884	33.76 [32.15–35.35]	8,706,163.84	0.48%	−4.17%	0.1304
65–74 years	199,857	33.41 [31.01–35.81]	6,677,222.37	192644	33.59 [32.91–34.27]	6,470,911.96	0.54%	−3.09%	0.2207
>74 years	196,258	33.98 [31.52–36.44]	6,668,846.84	183055	34.44 [33.79–35.09]	6,304,414.20	1.35%	−5.46%	0.1403
Sub-total	990,649		34,111,069.10	938,700		32,506,859.59		−4.70%	
Average/mean cost of an emergency episode		34.43			34.63		0.57%		0.1351
		[31.89–36.96]			[32.78–36.48]				
Average cost per acute/emergency claimant		126.63			149.88		18.36%		0.01023
		[122.70–130.56]			[143.92–155.84]				
Grand total	1,074,611		35,783,849.99	1,011,212		33,957,143.20		−5.10%	
Average cost of hypertensive episode (ACPE)		33.30			33.58		0.84%		0.1391
		[30.51–36.09]			[30.67–36.49]				
Average cost per hypertensive claimant (ACPC)		122.81			145.69		18.64%		0.01042
		[119.30–126.32]			[141.5–149.88]				

*CFU* Chronic follow-up episode, *EAE* Emergency Acute Episode, *ACPE* Average cost per episode.

*p-value for independent t-test for differences in pre-pandemic and pandemic ACPE and ACPC.

The change in health service use patterns for hypertension was measured in terms of EER, FER, EFER, and ECR with results shown in Tables 2 and 4. The EER and FER for the pre-pandemic and pandemic indicate an increase in the proportion of emergency/acute episodes in the pandemic year. The EFER for the pre-pandemic year was 11.80, increasing to 12.95 in the pandemic. The ECR in the pandemic year was 4.34, a 17.6% (95% CI: 17.3.0–17.9%; *p* < 0.001) increase from the reference year. Using the EER and ECR as a marker of hypertension severity, there was a noted increase in severity in the pandemic year, however, this was mostly due to an increase in ECR.

**Table 4:** Health Service use patterns for hypertension in the pre-pandemic and pandemic year.

	Pre-pandemic year	Pandemic Year	Difference (95% CI, *p*-value*)	Percentage change (comparing pandemic year to pre-pandemic)
Follow-up episode ratio (FER)	0.0781 (0.0776–0.0786)	0.0717 (0.0712–0.0722)	0.0064 (−0.0071–−0.0057, *p* < 0.001)	−8.20%
Emergency acute episode ratio (EER)	0.9219 (0.922–0.923)	0.9283 (0.928–0.929)	0.0064 (0.0057–0.0071, *p* < 0.001)	0.69%
(Emergency acute episode to follow-up episode ratio (EFER)	11.8	12.95	1.15	9.75%

*p-value for two proportions z-test.

### Financial impact of changes in service use patterns

The financial impact of the change in service use on the ACPC during the pandemic year was assessed. The change in ECR had the greatest impact on the ACPC during the pandemic year. The change in ECR accounted for 17.64% of the 18.64% increase in the ACPC in the pandemic year. The change in EER and FER together accounted for 0.28% of the change in ACPC. The financial impacts of changes in service use patterns on the cost of hypertension management per claimant are outlined in Table 5.

**Table 5:** The financial impact of change in service use patterns in pandemic year on Average cost per claimant (ACPC).

Factor/Ratio	Financial impact
Episode to claimant rate (ECR)	17.64%
Emergency episode ratio (EER)	0.66%
Follow-up episode ratio (FER)	−0.38%
FER + EER	0.28%

## Discussion

We examined the impact of COVID-19 on hypertension severity and hypertension-related health service utilisation patterns and claim expenditures using health insurance claim data obtained from Ghana’s NHIA. Our study found that the pandemic may have affected health-seeking behaviour for hypertension. There was an estimated 20% decline in the number of claimants who sought care for hypertension during the pandemic (target period) compared to pre-pandemic (reference period). This finding is parallel to those from other studies, both quantitative and qualitative, that found a decline in health-seeking behaviours [23–25] among Ghanaians during the pandemic. Several reasons that may explain this change in health-seeking behaviour have been highlighted by various authors [23–25]. Prime among these is the fear or concern of contracting COVID-19 infection at healthcare facilities. From the qualitative study by Abraham et al. [25]. Ghanaian adults with hypertension and diabetes refrained from seeking healthcare during the pandemic as they not only believed that having these NCDs made them more susceptible to COVID-19 but that being infected would exacerbate these chronic conditions resulting in severe complications or even death. Such fears are not unfounded; Pavey et al. [26] have reported that hypertension is associated with a 22% increase in the odds of severe COVID-19 infection in the UK population and other studies [27, 28] have observed an association between a history of COVID-19 infection and an increase in blood pressure. It may be further argued that lockdown policies and other measures taken during the pandemic, as well as the closure of some health facilities and the scarcity of health workers, may have impeded access to healthcare access for some patients who may have sought care [25].

Although there was a significant drop (20%) in the number of individuals who sought care for hypertension during the pandemic year, the average cost per claimant (ACPC) increased. Resultingly, the change in the total cost for all hypertension claims in the pandemic year was disproportionate to the decline in the number of claimants, falling by only 5.1%. The increase in the ACPC can mostly be attributed to the increase in ECR, a proxy for hypertension severity. Thus, although fewer people sought care for hypertension in Ghana during the pandemic, those who did, required more episodes of care possibly due to increased disease severity. This study also showed, from the change in EER, that a marginally higher proportion of hypertensive episodes in the pandemic year were for emergency/acute care. It may be inferred from these findings that COVID-19 and its associated infection preventive measures may have led to the worsening of hypertension in Ghana. These findings on increased episode intensity of hypertension are congruent with findings from other studies (in other settings) that examined the impact of COVID-19 on hypertension. For example, Gotanda et al. [29] in their time series analysis found an increase in blood pressure and poor disease control among individuals with hypertension in the US during the pandemic. Singh et al. [30] also observed a worsening of hypertension and diabetes in India during the pandemic, with pandemic-related factors such as job/income loss and difficulties in accessing medications due to movement restrictions being associated with the worsening of these NCDs.

Findings from the present study also showed that for both the pre-pandemic and pandemic years, more than 90% of claims for primary hypertension were for emergency care. Additionally, emergency/acute care accounted for over 95% of total hypertension claim costs. While a stark finding, this is perhaps not surprising. The Ghana Demographic Health Survey showed that among Ghanaians aged 15–49, about 63% of women, and 86% of men with high blood pressure were unaware that they did and only 17% of women and 6% of men with hypertension are on medication and had the condition well controlled. Given this and the insidious and silent progression of hypertension [16, 17], most claimants may only be presenting to health facilities when the disease becomes evident due to severe or life-threatening symptoms. Such presentations require emergency/acute care which is more resource-intensive leading to increases in ACPC and the overall hypertension-related costs the NHIS incurs.

### Policy implications

The second edition of Ghana’s national NCD policy [31] framework published in 2022 outlined interventions to address NCDs such as hypertension; however, the policy fails to acknowledge and address the effect of the COVID-19 pandemic on NCDs and how NCD interventions and policies need to be adapted in the wake of the pandemic. This study provides relevant considerations for policymakers in Ghana within this context.

Firstly, the drop in hypertensive claimants and health-seeking behaviour during the pandemic indicates forgone or delayed health care for individuals with hypertension in the country. Therefore, policymakers need to consider interventions that ensure the bridging of this gap in healthcare to delay disease progression and prevent adverse complications. Further, given the impact of emergency/acute care on the NHIS expenditure, preventive interventions and early diagnosis are necessary to not only reduce this financial impact but also to reduce disease burden and other related outcomes such as productivity losses. This is particularly important for the NHIS which faces financial sustainability challenges [32]. Reducing the volume of emergency care for hypertension, which from this study is shown to cost 70% more than follow-up care, could contribute to improving the long-term sustainability of the scheme.

### Limitations

This study used NHIS claim data from only 3 out of the 16 regions in Ghana. Though the three regions were selected to be representative of the three geographic belts in Ghana, the impact of the pandemic was nationwide. This means the estimated impact of the pandemic on hypertension may not have been fully captured at a national level.

As the NHIS claims data are classified using diagnostic-related grouping codes, a possible undercount of hypertension episodes may occur if complications arising from hypertension such as kidney disease may have been classified differently or misclassified without indicating hypertension as a primary diagnosis. Also, given the limited variables in the NHIA dataset, relevant factors such as socioeconomic status and comorbidities that are associated with hypertension severity [33–35], could not be controlled for in this study.

Finally, while this study examines the change in service patterns during the pandemic year, the comparison is to only one reference year (pre-pandemic year (03/2019–02/2020). It may have been more beneficial to establish the usual year-on-year trends in NHIS hypertension service use patterns several years before the pandemic and have that as a reference. However, such analysis was out of the scope of this study.

## Conclusion

This study highlights the impact of the COVID-19 pandemic on disease severity and health service use patterns for hypertension. While there was a significant drop in the number of claimants for hypertension during the pandemic, this was associated with increased severity and healthcare costs as individuals who sought care for hypertension required more frequent and more expensive care. Findings from this study would be relevant in building strategies and for policymaking on NHIS and interventions for hypertension and other NCDs in Ghana (and comparable countries in SSA).

## Summary

### What is already known about the topic

- Hypertension is a leading cause of mortality and morbidity in Ghana and other sub-Saharan African countries.
- It is acknowledged that the COVID-19 pandemic and its associated measures have disrupted healthcare for hypertension and other NCDS. However, limited studies have explored the impact of these disruptions in Ghana and other sub-Saharan African countries where healthcare systems were already fragile.

### What this study adds

- This study contributes to the literature by examining the impact of the pandemic on the management of hypertension in Ghana, with a focus on disease severity, health service use patterns, and expenditures, using claims data from Ghana’s National Health Insurance Authority.
- This study found significant changes in hypertension management in Ghana during the pandemic year. Although there was a 20% reduction in the number of hypertension claimants, there was an increase in disease severity and the average cost per hypertension claimant.

## Data Availability

Data used for the analysis of this study is publicly accessible upon request from the Ghana National Health Insurance Authority
